# The Scientific Response to a Pandemic 

**DOI:** 10.1371/journal.ppat.0020009

**Published:** 2006-02-24

**Authors:** Gigi Kwik Gronvall, Richard E Waldhorn, D. A Henderson

Tomorrow, the world could face a pandemic. It could be due to H5N1 avian influenza—experts warn that it is just a matter of time—or a different influenza strain, an unknown pathogen, or a bioterrorist attack. In all cases, biological scientists face the challenge of characterizing the pathogen and determining how to control it. Their scientific judgments and public statements will shape the global pandemic response. During the SARS outbreak in 2003, scientists generated information that influenced everything from medical treatments to travel restrictions, trade policy, and political decisions. Given the importance of getting scientific information out into the world, scientists should consider now how they will respond and communicate in the setting of the next pandemic.

In hopes of sparking that discussion, the Center for Biosecurity at the University of Pittsburgh Medical Center organized an International Conference on Biosafety and Biorisks (http://upmc-biosecurity.org/pages/events/biosafety/report.html) in collaboration with the World Health Organization (WHO) Communicable Disease Surveillance and Response Office in 2005. More than 150 scientists and public health practitioners from 25 countries gathered in Lyon, France, to hear speakers from the WHO, the European Commission, scientific journals, Interpol, and public health networks—many of the institutions and individuals who will likely play key roles in the global response to the next pandemic. By discussing the biosafety and biosecurity challenges presented by past epidemics such as SARS, participants recognized the importance of scientific and public health collaboration in combating disease—and the need to plan.

Each epidemic is different, and will have its own scientific and political dimensions that make planning difficult. However, based on past epidemics, there are some likely patterns. Researchers will need to share biological samples between laboratories, sometimes internationally; decision makers and journalists will want the latest information, which may not be peer reviewed; and researchers will risk contracting the disease they research, which could then spread outside of the laboratory. Scientists need to harmonize and modernize standards and training in these areas, to help make their response to a pandemic prompt, accurate, and safe.

## Access to Biological and Clinical Samples

Scientists need access to samples from patients and laboratories in order to conduct research and public health surveillance, as well as to develop diagnostic tests. Not every researcher who requests a strain for research in the midst of a pandemic is likely to get it, but international standards for documenting, referencing, tracking, and shipping samples would save valuable time in a pandemic.

For example, during the SARS outbreak in 2003, laboratories with access to samples were not always willing to share with other, potentially competing, laboratories. Shipping samples was also a problem. In one report, “It took eight weeks to determine the protease structure [of the SARS coronavirus]. But half that time was taken in obtaining the DNA for the viral proteins from collaborators…and getting it through customs” [1]. Sharing samples can be difficult even within the same research institution. Many clinical laboratories cannot release patient samples without preapproved protocols from an Institutional Review Board. Universities should consider how to make it easier for their researchers to comply with Institutional Review Board regulations [2], including developing emergency Institutional Review Board protocols.

H5N1-sample access problems have already begun due to slow responses for shipping samples to international reference laboratories. Noted virologists from universities outside of the WHO laboratory network have also had difficulty getting access to samples and thus contributing their expertise [3]. Bioterrorism concerns have added to the problem, as Japan and Hong Kong require permits for handling highly pathogenic viruses, including H5N1 [3]. In the US, H5N1 is a select agent, so the Department of Agriculture must certify laboratories before they can receive the virus [4]. However, improved standards for tracking samples, if correctly applied, could bring benefits to biosecurity and also help researchers. According to a WHO official, “During the [SARS] outbreaks, lots of samples were taken...and we don't know where they all are” [5]. SARS may no longer be infecting people naturally, but there is risk that a laboratory sample could end up in improperly trained or malevolent hands. H5N1 could be similarly dangerous. In a time when one can track a package from warehouse to mailbox, the current methods for keeping track of vital biological samples are grossly out of date.

## Standards for Communication of Scientific Results

Scientists, journal editorial boards, and other scientific professional organizations should consider creating standards for communicating scientific results, so decision makers can make use of the information and scientists can get professional recognition for their work. Traditionally, scientists communicate results through conferences and peer-reviewed publications. However, during the SARS epidemic, it became common to announce results in a press conference. Sharing results with the press may save time, but there are problems with this approach: the reports are not peer reviewed and newspapers do not usually give in-depth technical reports of experimental results, which limit the usefulness of news reports to researchers.

Publication in scientific journals also poses problems. While journals provide peer review and professional recognition for the authors, publication is often slow. In the absence of hard data, some reporters cover the reactions of people who have seen papers under review—for example, “Researchers who have seen [the] data say they are convinced” [6]—rather than the facts themselves. One way to speed dissemination of results is for scientific journals to agree to peer review articles of importance within 24 hours, before Web posting. Reviewers should also have strict deadlines so that they do not delay important information being made public.

## Biosafety Standards

Accidents happen, and working with infectious agents will never be risk free. However, a lack of training, mentoring, and formal standards for biosafety leaves researchers at unnecessary risk of infection, and has had tragic effects outside of the laboratory. There are no international standards for biosafety, but there are guidelines from the WHO and the Centers for Disease Control and Prevention that could be developed into formal training for all researchers.

The consequences of not adhering to laboratory biosafety guidelines can be dire. Some experts believe that the dominant strain of flu in 1977 originated from a laboratory [7]. During the SARS outbreak, there were four documented laboratory accidents in three laboratories— Singapore, Taiwan, and China [8]. In each case, the laboratories had the best equipment, but training and experience with pathogens were lacking. The public may not easily forgive the scientific community at large for an epidemic that starts in a laboratory. The scientific community has a responsibility to take reasonable precautions to prevent accidents and to train researchers in safety. Accidents should be documented, and the lessons learned from those accidents should be incorporated into safety training. If scientists do not promote biosafety, regulations could be imposed on them. The strict safety regulations proposed for laboratories in Boston, for example, directly result from three laboratory-acquired cases of tularemia [9].

## Global Public Health Relies on Networks

In the event of an avian flu pandemic, it is likely that the WHO will coordinate the international response, as they did with SARS. However, no one organization has the resources and expertise to deal with all of the scientific and medical complexities that a pandemic will present. The WHO relies on a variety of information networks and laboratories, such as the Global Public Health Intelligence Network, which gathers reports of disease outbreaks in seven languages, and proMED-mail, which is an open-source electronic reporting system for disease outbreaks. A similar set of open-source electronic systems for scientists to convey information and collaborate could also amplify WHO scientific expertise and the availability of hard data for decision making in a pandemic.

## Starting to Plan

Scientific information fuels and directs the response to epidemics. Public health professionals, clinicians, politicians, journalists, and members of the public will make critical decisions based on what is known about a disease as an outbreak unfolds. Scientists can take this opportunity, before the next pandemic, to plan to give accurate information to those who need it as fast and as safely as possible.

## 

**Figure:**
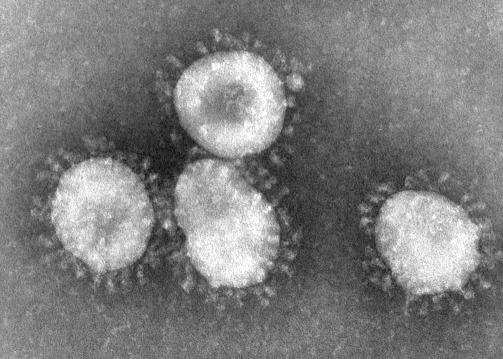
A coronavirus, with its distinctive halo or crown-like appearance, is recognized as the etiologic agent of the 2003 SARS outbreak (Image: F. Murphy, CDC)

**Figure:**
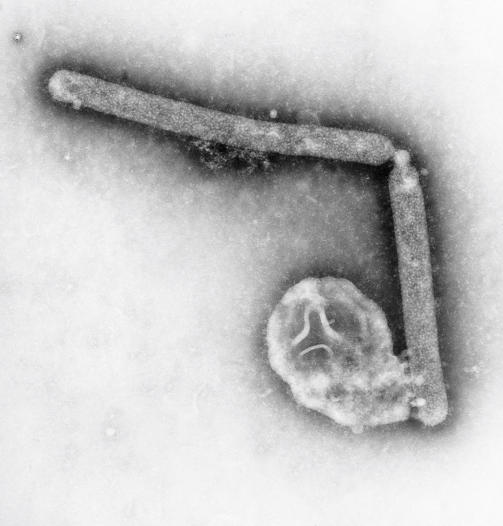
Transmission electron micrography (108,000×) reveals two avian influenza A (H5N1) virions (Image: C. Goldsmith, J. Katz, CDC)

## Abbreviation

WHO: World Health Organization
